# Can essential fatty acids (EFAs) prevent and ameliorate post-COVID-19 long haul manifestations?

**DOI:** 10.1186/s12944-024-02090-4

**Published:** 2024-04-19

**Authors:** Undurti N. Das

**Affiliations:** 1 UND Life Sciences, 2221 NW 5th St, Battle ground, WA 98604 USA; 2 Department of Biotechnology, Indian Institute of Technology-Hyderabad, Sangareddy, Telangana, India; 3 Department of Immunology and Rheumatology, Arete Hospitals, Gachibowli, Hyderabad, 4500032 India

**Keywords:** SARS-CoV-2, COVID-19, Essential fatty acids, Eicosanoids, Lipoxin A4, Resolvins, Protectins, Maresins, Polyunsaturated fatty acids, Inflammation

## Abstract

It is hypothesized that COVID-19, post-COVID and post-mRNA COVID-19 (and other related) vaccine manifestations including “long haul syndrome” are due to deficiency of essential fatty acids (EFAs) and dysregulation of their metabolism. This proposal is based on the observation that EFAs and their metabolites can modulate the swift immunostimulatory response of SARS-CoV-2 and similar enveloped viruses, suppress inappropriate cytokine release, possess cytoprotective action, modulate serotonin and bradykinin production and other neurotransmitters, inhibit NF-kB activation, regulate cGAS-STING pathway, modulate gut microbiota, inhibit platelet activation, regulate macrophage and leukocyte function, enhance wound healing and facilitate tissue regeneration and restore homeostasis. This implies that administration of EFAs could be of benefit in the prevention and management of COVID-19 and its associated complications.

## Introduction

So called “long-haul syndrome” following acute SARS-CoV-2 infection and/or post-COVID-19 vaccination is not uncommon. It is estimated to occur in about 10–20% of subjects who have had SARS-CoV-2 infection and/or COVID vaccination. The long-haul syndrome manifestations include fatigue, post-exertional malaise, memory loss, and other neurocognitive impairments that may last from months to years [1, 2]. It is also known that some of these patients may have several cardiac manifestations in the form of cardiac arrhythmias, heart failure, unexplained hypotension, and sometimes sudden cardiac death. Neurological manifestations may include memory impairment, inability to concentrate, and sometimes stroke. Some of the hypotheses put forward to explain the long-haul syndrome include: (i) continued presence of the virus or viral particles such that they are able to stimulate the immune system leading to tissue damage; (ii) there could occur auto-antibody production and pro-inflammatory cytokine(s) secretion resulting in chronic inflammation [2] and (iii) both features of i and ii [2, 3]. Post-COVID long-haul syndrome has been found to be associated with the presence of platelet dysfunction and hypercoagulability state and endothelial dysfunction [4]. Autonomic nervous system dysfunction may also occur [5–9] that could explain some, if not all, the features of long-haul syndrome [10–12]. It is likely that all the mechanisms proposed may occur in different subsets of patients. But what is not clear is how and why the features of long-haul syndrome occur.

### Features of long- COVID

In a study performed in 275 individuals, it was found that those with long COVID had significantly higher levels of non-conventional monocytes (CD14^low^CD16^high^), elevated expression of MHC class II (HLA-DR), lower circulating populations of conventional type 1 dendritic cells (cCD1), increase in the absolute count of double negative B cells, absolute count of CD4^+^ cells with a lower number of circulating CD4^+^ central memory cells (CD45RA^−^ CD127^+^CCD7^−^) with an absolute increase in the number of exhausted CD4^+^ T cells [12]. Stimulated CD4^+^ cells produced significantly higher levels of intracellular IL-2, IL-4 and IL-6 in those with long COVID. Surprisingly, no significant increase in the production of IFN-γ, IL-17 and TNF was noted in long COVID. Those with long COVID showed higher levels of SARS-CoV-2 antibody titers especially anti N IgG levels compared to the control. They also had significantly lower plasma cortisol levels in association with increased complement C4b, CCL19, CCL20, galactin-1, CCL4, APRIL and LH and decreased IL-5 with no significant change in the plasma adrenocorticotropic hormone (ACTH) levels compared to control. This suggests that the hypothalamic–pituitary–adrenal axis response is abnormal. Several reports implicated a role for G-protein coupled receptor (GPCR) antibodies in the pathogenesis of long COVID [13, 14] that particularly targeted β-adrenergic receptors and angiotensin-II receptor. The presence of GPCR antibodies may explain autonomic dysfunction seen in those with long COVID. These patients may also have higher IgG antibody levels against total anti-S and anti-receptor binding domain (RBD), distinct regions of specific spike protein and SARS-CoV-2 (especially against linear peptides) compared to control [12]. In addition, these patients showed elevated antibody titers against several herpes antigens, Epstein-Barr virus (EBV) and the varicella zoster virus (VZV) glycoprotein implying that they have hyperimmune responses not only to SARS-CoV-2 but also to some latent viral infections. Such specific abnormal antibody responses in long COVID subjects may explain as to why G-protein coupled receptor (GPCR) antibodies particularly targeting β-adrenergic receptors and angiotensin-II receptor occur in them. The presence of persistent SARS-CoV-2 viral antigens, reactivation of latent viral infections, autoimmunity, dysbiosis, tissue damage and resultant chronic inflammation seen in long COVID implies skewed T-helper-2-cell CD4^+^ T cell activation [12]. But it is not clear whether the diverse biological features seen in long COVID are solely based on the peripheral immune factors or secondary to organ-system-specific dysfunctions.

### Serotonin deficiency in “long-haul syndrome”

**Fig. 1:**
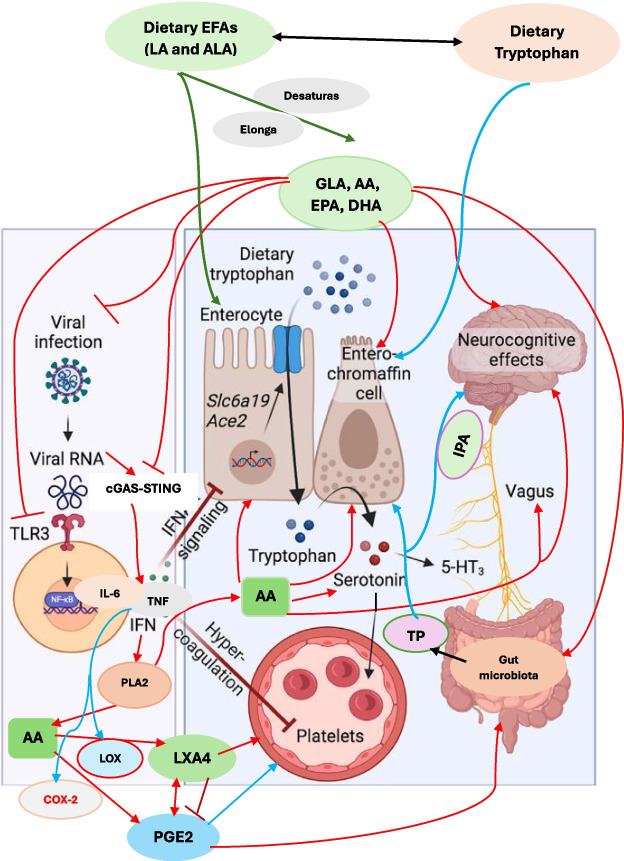
Scheme showing potential interaction between EFAs and their metabolites and serotonin. Legend to Figure 1. Indoles are synthesized from tryptophan by gut microbiota that express tryptophanase (TP). Indoles are cytoprotective molecules. Indolepropionic acid (IPA) synthesized by gut microbiota is a neuroprotective substance and binds to the pregnane X receptor (PXR) in intestinal cells, to facilitate mucosal homeostasis. IPA absorbed from the gut is transferred to the brain to prevent β-amyloid fibril formation. Tryptophan is metabolized to indole-3-aldehyde (I3A) by gut microbiota that acts on the aryl hydrocarbon receptor (AhR) in intestinal immune cells. Gut microbiota have the ability to alter the expression of serotonin-related genes and thus, alters its (serotonin) biosynthesis. Gut microbiota (i) directly act on enterochromaffin (EC) cells to increase colonic tryptophan hydroxylase 1 (Tph1) expression and promote serotonin synthesis; (ii) alter host by direct action or through their metabolites, short chain fatty acids (SCFAs), tryptophan, tryptamine, and secondary bile acids; (iii) SCFAs stimulate serotonin synthesis and release by their action on enterochromaffin cells; (iv) influence serotonin metabolism; and (v) promote Tph1 expression and stimulate serotonin synthesis (90, see Fig. 3) (tryptamine is a ligand for the 5-HT_4_ receptor (5-HT_4_R) and secondary bile acids, formed by the gut microbiota). Gut microbiota survival, proliferation and metabolism are influenced by EFAs. Dietary EFAs are converted to their long-chain metabolites by desaturases and elongases. EFAs and their metabolites are necessary for the integrity and function of enterochromaffin cells and gut function. By their action on enterochromaffin cells, EFAs can influence serotonin metabolism. EFAs and their metabolites can minimize TLR3 expression. Similarly, EFAs and their metabolites suppress cGAS-STING pathway and inhibit the production of pro-inflammatory cytokines and thus, inhibit their production. The coagulation by aggressive platelets aggregation can also be suppressed by EFAs and their anti-inflammatory metabolites and thus, prevent thrombotic episodes. EFAs and their metabolites stimulate vagus nerve and thus enhance the production of acetylcholine that has anti-inflammatory actions. EFAs and acetylcholine enhance the production of LXA4, a potent anti-inflammatory bioactive lipid (derived from AA). Thus, EFAs and their metabolites have a plethora of actions that explain their role in post-COVID long haul syndrome. The red arrows indicate pro-inflammatory pathways. The blue arrows indicate the pathways stimulated by cytokines that are pro-inflammatory in naure. The green arrows indicate the metabolism of dietary EFAs to their long-chain metabolites and action on enterocytes and their beneficial actions

A recent study [5] reported that SARS-CoV-2 induced COVID-19 is associated not only with type I interferon (IFN)-driven inflammation but also reduced plasma serotonin levels. This has been attributed to diminish in the intestinal absorption of tryptophan (the serotonin precursor); reduced serotonin storage due to platelet hyperactivation and reduced platelet count; and/or enhanced activity of MAO (monoamine oxidase). The decreased serotonin levels reduce the activity of the vagus nerve that, in turn, results in impairment of hippocampal responses and memory (see Fig. 1) that led to the suggestion that reduced serotonin may underlie the cause of long COVID manifestations. If this is true, it implies that enhancing serotonin levels could be of benefit in the management of long COVID. But it is not clear whether decreased serotonin levels are the cause or effect of long COVID.

I propose that the serotonin abnormalities [5], reactivation of latent viral infections, autoimmunity, dysbiosis and impaired tissue repair resulting in chronic inflammation and associated skewed T-helper-2-cell CD4^+^ T cell activation [12] in long COVID-19 are secondary to altered or defective metabolism of essential fatty acids (EFAs).

### Metabolism of EFAs

**Fig. 2:**
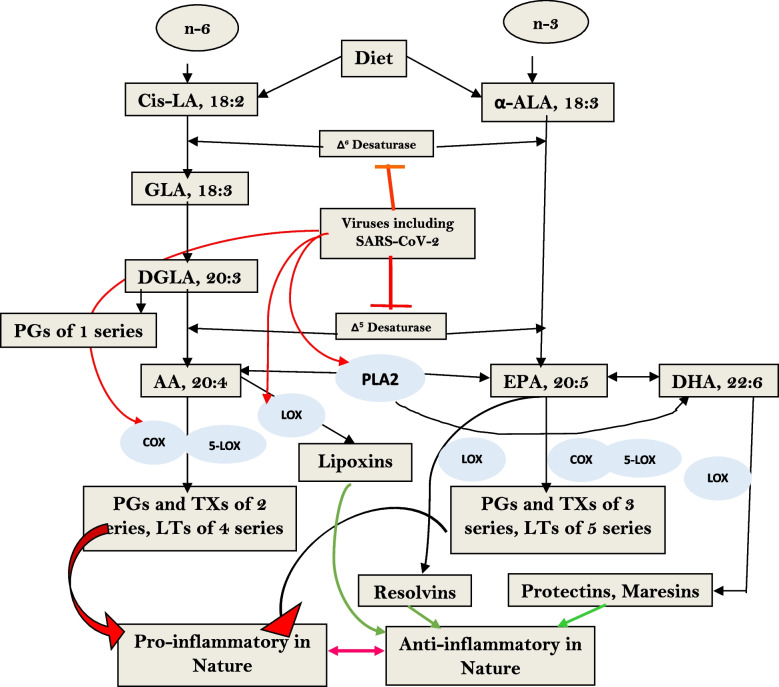
Metabolism of essential fatty acids (EFAs), their role in inflammation and factors that influence desaturases. SARS-CoV-2 activates PLA2 (phospholipase A2) and induces the release of GLA, DGLA, AA, EPA, and DHA from the cell membrane lipid pool. SARS-CoV-2 inhibits the activities of desaturases and thus, decreases the formation of GLA, DGLA and AA from LA and EPA and DHA from ALA. SARS-CoV-2 activates both COX and LOX enzymes resulting in increased formation of PGs (prostaglandins), LTs (leukotrienes) that have pro-inflammatory action and. enhanced formation of LXA4, resolvins, protectins and maresins that may induce hypotension

Dietary linoleic acid (LA, 18:2 n-6) and alpha-linolenic acid (ALA, 18:3 n-3) are considered as essential fatty acids (EFAs) and they are crucial for our survival. EFAs are metabolized by enzymes desaturases and elongases to form their respective metabolites that have many biological actions (see Figs. 1 and 2). EFAs and their metabolites have a regulatory role in producing immune response, serve as endogenous antibiotics, and have cytoprotective and genoprotective actions and are needed for wound healing [15–20]. They also serve as local hormones in general to modulate the actions of several physiological factors. EFAs behave as neurotransmitters and maintain the gut microbiota and its metabolism. The diversified actions and interactions of EFAs during different physiological & metabolic activities is critical for retaining the functional homeostasis. EFAs also modulate the concentrations of cAMP, cGMP, activate several cell surface G protein-coupled receptors (GPCRs), influence biological processes by regulating peroxisome proliferator–activated receptors, sterol regulatory element binding protein 1, Toll-like receptor 4, G protein–coupled receptors, and other putative mediators and serve as signaling molecules and stimulate or inhibit DNA transcription [21–25]. In addition, AA and other fatty acids function as mechanotransducers to convey messages from the extracellular environment through the cell membrane to the nucleus to regulate gene expression and PI3K/Akt/mTOR pathway that is involved in many physiological and pathological processes, and thus, regulate the normal cellular events. It is noteworthy that AA and other fatty acids are potent regulators of the expression and levels of NF-κB and IκB, TNF-α, IL-6 and cGAS-STING pathway [15, 19, 26–40]. In addition, AA and PUFAs regulate the gene expression [40], but peroxide products of the same could lead to cause the oxidative stress, inflammation, atherosclerosis, cancer, and other disorders. The EFAs play an integral role in regulating the cellular activities and its effectiveness is influenced with factors such as, Magnesium, folic acid, vitamins B1, B12, B6, and C; calorie restriction, adequate protein intake, and insulin for optimum metabolism. They serve as a co-factors for the activity of desaturases that are needed for the conversion of dietary EFAs, LA and ALA to their respective long chain metabolites. GLA, DGLA, AA, EPA and DHA, which are precursors of several beneficial metabolites such as PGE1 (from DGLA), PGI2 (from AA), lipoxin A4, resolvins, protectins amd maresins (from AA, EPA and DHA) that have potent anti-inflammatory and immunoregulatory actions [15]. In contrast, high glucose, saturated and trans fatty acids, cholesterol, and high fat diet inhibit the activities of desaturases and enhance the formation of pro-inflammatory eicosanoids [15, 20]. It is noteworthy that, supplementation of EFAs do not have any significant side effects. In view of their immunomodulatory and pro-/anti-inflammatory actions, there is a theoretical possibility that excess consumption of EFAs may result in immunosuppression especially in the elderly and immunocompromised individuals.

### EFAs and serotonin

EFAs and their metabolites regulate the synthesis and actions of serotonin. Supplementation of EFAs modulate central nervous system (CNS) serotonin and dopamine metabolism and impulsive behaviors related to them [41]. An inverted U-shaped curve relationship exists between serotonin binding, AA and depression severity [42], suggesting that AA status has an impact on the pathophysiology of depression. AA modulates serotonin transport implying that EFAs are of therapeutic benefit in the treatment of major depression.

EPA increases serotonin release from presynaptic neurons by reducing PGE2 levels whereas DHA influences serotonin receptor action by increasing cell membrane fluidity [43]. These results suggest that EPA/DHA (and possibly, AA) deficiency results in dysfunctional serotonin activation and function that contributes to neuropsychiatric disorders and depression. This proposal is interesting in the light of the observation that AA and DHA have a role in exocytosis of neurotransmitters [44–50]. This implies that AA/EPA/DHA have a critical role in the exocytosis of various neurotransmitters and their deficiency can lead to the development of various neuropsychiatric disorders including those related to post-COVID and post-COVID-vaccine long haul syndrome.

Impulsive violence, suicide, and depression are strongly associated with low concentrations of cerebrospinal fluid 5-hydroxyindoleacetic acid (CSF 5-HIAA). Low plasma polyunsaturated fatty acids predicted both CSF 5-HIAA and CSF HVA concentrations implying that EFAs influence CNS serotonin and dopamine metabolism and thus, play a role in neuropsychiatric conditions [41]. These results are in support of the contention that low concentrations of serotonin reported in those with long-haul or long haul or long COVID could be attributed to deficiency of EFAs (such as AA/EPA/DHA).

In view of the close interaction(s) between EFAs and their metabolites and serotonin metabolism, it is likely that SARS-COV-2 may affect tryptophan metabolism and 5-HT release and related dysbiosis. This implies that 5-HT associated prothrombotic events due to platelet activation/aggregation seen in those with acute SARS-CoV-2 infection and long haul COVID syndrome may also be secondary to alterations in EFAs metabolism. This is so since GLA/DGLA/AA/EPA/DHA and their metabolites can alter platelet function and thrombosis by virtue of their actions on platelets and serotonin metabolism. It is suggested that SARS-COV-2 associated hypercoagulant events that may be dependent or independent on 5-HT are secondary to deficiency of EFAs and their anti-platelet metabolites. The observation that elevated PF4 autoantibody in relation to heparin-induced thrombosis reported in SARS-CoV-2 infection and long haul COVID may also be due to a deficiency of EFAs and their immunomodulatory metabolites.

### EFAs in COVID-19

Previously, I proposed that EFAs deficiency, especially that of AA/EPA/DHA, could lead to increased susceptibility and/or progression of COVID-19. This hypothesis is based on the observations that EFAs and their long chain metabolites AA/EPA/DHA have the ability to inactivate several enveloped viruses including SARS-CoV-2. EFAs metabolites prostaglandin E1 (PGE1), lipoxin A4 (LXA4), resolvins, protectins and maresins enhance phagocytic capacity of macrophages and leukocytes at the site of inflammation and clearing the cell debris near injury rapidly to enhance wound healing. Furthermore, EFAs and their metabolites regulate vasomotor tone, inflammation, thrombosis, immune response, T cell proliferation and secretion of cytokines, stem cell survival, proliferation and differentiation, and leukocyte and macrophage functions, JAK kinase activity and neutrophil extracellular traps- actions that may explain their (EFAs-AA/EPA/DHA) potential critical role in the pathobiology of COVID-19 [51–54]. This concept is supported by the fact that LA, AA, and EPA stabilize the locked confirmation and inhibit the replication of SARS-CoV, MERS-CoV, SARS-CoV-2, and their variants [55, 56]. SARS-CoV-2 virus induces marked perturbation in the metabolism of LA and AA; and supplementation of LA or AA to SARS-CoV-2 and similar viruses infected cells led to marked suppression of the virus replication. These results suggest that host EFAs metabolism plays a significant role in the pathogenesis of COVID-19 and other viral diseases suggesting that these fatty acids are likely to be of significant benefit in their prevention and management.

### EFAs influence gut microbiota and vice versa

The human gut is teaming with millions of bacteria. These bacteria are passed on to the fetus at the time of birth, some are obtained through breast feeding and some are consumed along with food. These microorganisms that form the gut microbiota can have both beneficial and harmful actions [57]. The ‘gut microbiota’ not only co-evolved with the host (humans) over thousands of years but also has an intricate and mutually beneficial relationship [58–60].

Short-term dietary changes produce rapid but reversible shifts in the gut microbiota composition, while longer-term changes induce changes in the genomic composition and metabolic activities of microbiota. Diet is one major factor that influences the gut microbiota composition, metabolism, and genetic architecture. On the other hand, changes in the gut microbiota have both direct and indirect effects on gastrointestinal architecture, function, and metabolism. The ability of gut microbiota to produce acetate, butyrate, and propionate (SCFAs-short chain fatty acids) depends on the diet that is consumed by the host, whereas gut microbiota SCFAs and other metabolites affect several physiological processes and pathological conditions [61–66]. Human lifestyle and geographical differences including diet or cooking practices beget gut microbiota differences. This may explain differences in gut microbiota of individuals consuming plant-based diets compared with those who consume high-meat and high-fat Western diets [67] that, in turn, may reflect the differences in the susceptibility to various diseases, their response to treatment and prognosis. This is particularly true of individual differences in gut microbiota depending on their lifestyle, diet, geography, method(s) of dietary preparation, cooking and various components used in cooking. Thus, gut microbiota is highly plastic in its composition, function, and properties [68]. One such factor that is less well appreciated is the effect of unsaturated fatty acids on the composition and functions of gut microbiota.

Both n-6 and n-3 fatty acids alter the diversity and abundance of the gut microbiota that, in turn, affect the metabolism and absorption of these fatty acids. Both EFAs and gut microbiota interact with each other and produce their effect(s) on inflammation, obesity, and metabolic diseases by altering the intestinal wall integrity and host immune cells [69–73]. Furthermore, human gut microbiota converts host diet into numerous bioactive metabolites [74–78]. It is noteworthy that gut microbiota has the unique ability to convert EFAs such as LA into various intestinal fatty acid isomers that regulate the mucosal immune system of the host and enhance the formation of CD4^+^ intraepithelial lymphocytes. In a similar fashion, gut microbial derived lipid mediators {such as 9,10-dihydroxy-12Z-octadecenoic acid (9,10-DiHOME) and all-*trans* retinoic acid (atRA)} and SFAs can enhance the development of regulatory T (Treg)-cell activity and thus prevent the occurrence of inflammatory bowel disease (IBD). and other inflammatory conditions [75–78]. These results suggest that gut microbiota can convert dietary EFAs to other biologically active metabolites and EFAs influence gut microbiota and their metabolism that are ultimately responsible for their beneficial actions [74–80].

EFAs are involved in maintaining the various physiological and immunological mechanisms of the body, alongside their greater ability to act as antimicrobials and growth regulatory molecules. Hence, EFAs suppress the growth of pathogenic microbes of gut and promote the survival, proliferation, and metabolism of beneficial microbiota that produce SCFA metabolites (acetate, butyrate, and propionate) for essential physiological functions to build the protective immune response.

### Gut microbiota and serotonin

**Fig. 3:**
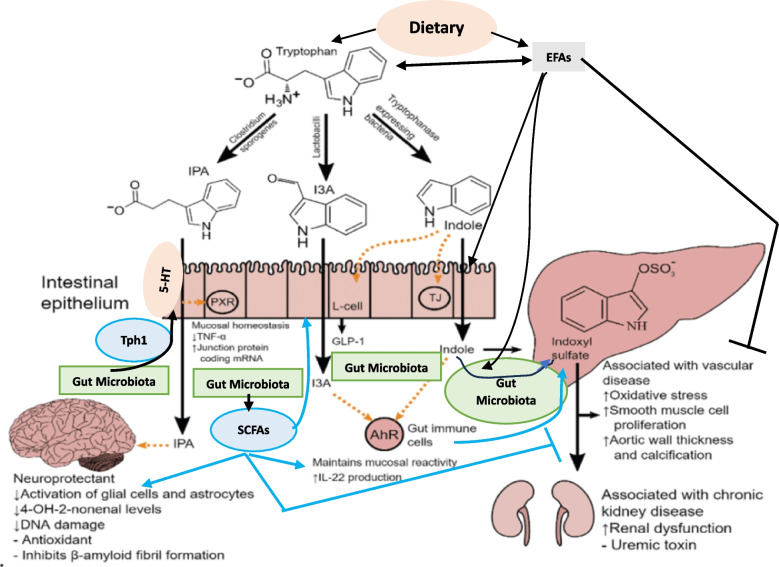
Scheme showing tryptophan metabolism and its interaction with gut microbiota and EFAs. Legend to Figure 3. Indoles are synthesized from tryptophan by gut microbiota that express tryptophanase. Indolepropionic acid (IPA) synthesized by gut microbiota is a neuroprotective substance that binds to several receptors, including the pregnane X receptor (PXR) in intestinal cells, to facilitate mucosal homeostasis. IPA is absorbed from the gut and transferred to the brain, where it prevents β-amyloid fibril formation. Tryptophan is metabolized to indole-3-aldehyde (I3A) by gut microbiota acts on the aryl hydrocarbon receptor (AhR) in intestinal immune cells. Gut microbiota act (i) directly on enterochromaffin (EC) cells to increase colonic tryptophan hydroxylase 1 (Tph1) expression and promote serotonin synthesis; (ii) alter host by virtue of their metabolites, including short chain fatty acids, tryptophan, tryptamine, and secondary bile acids; (iii) short chain fatty acids (SCFAs) stimulate serotonin synthesis and release by acting on enterochromaffin cells; (iv) tryptophan metabolism is regulated by the gut microbiota and thus, the gut microbiota influences serotonin metabolism; and (v) tryptamine is a ligand for the 5-HT_4_ receptor (5-HT_4_R) and secondary bile acids, formed by the gut microbiota promote Tph1 expression and stimulate serotonin synthesis [88].

The human gut produces approximately 95% of total body serotonin that has actions relevant to the functions of the gut and acts locally to activate afferent nerve endings that can influence the central nerve system. The gut-brain axis works to influence each other linking the enteric and central nervous systems that is not only anatomical, but it includes endocrine, humoral, metabolic, and immune effector cells and influence mood, cognition, and mental health. The gut-brain link is established by the autonomic nervous system, hypothalamic–pituitary–adrenal (HPA) axis, and enteric nervous system that allows the brain to influence intestinal activities. This gut-brain axis is influenced by the gut microbiota [81–83]. This gut-brain interaction explains as to why mood disorders, such as anxiety, depression, and autism spectrum disorders have links to functional gastrointestinal disruptions [84]. Gut bacteria influence fetal and neonatal neurologic development [85]. Diet also influences the gut microbiota and its impact on cognitive function [86]. Gut dysbiosis affects adult hippocampal (HPC) neurogenesis and can cause depression-like behavior, implying that communication between the gut and the brain may have a role in its (depression) development. It was reported that gut dysbiosis induces changes in both serotonin and dopamine neurotransmission pathways in the brainstem and hippocampus that could be abrogated by vagotomy suggesting that vagal afferent pathways have a role in gut microbiota-mediated effects on the brain [87], see Figs. 1 and 3).

It is noteworthy that the gut microbiota alters the expression of key serotonin-related genes and thus, alters its (serotonin) biosynthesis. Gut microbiota can act (i) directly on enterochromaffin (EC) cells to increase colonic tryptophan hydroxylase 1 (Tph1) expression and promote serotonin synthesis; (ii) alter host by virtue of their metabolites, including short chain fatty acids, tryptophan, tryptamine, and secondary bile acids; (iii) short chain fatty acids (SCFAs) stimulate serotonin synthesis and release by acting on enterochromaffin cells; (iv) tryptophan metabolism is regulated by the gut microbiota and thus, the gut microbiota influences serotonin metabolism; and (v) tryptamine is a ligand for the 5-HT_4_ receptor (5-HT_4_R) and secondary bile acids, formed by the gut microbiota promote Tph1 expression and stimulate serotonin synthesis (90, see Fig. 3). Gut microbiota induces maturation of enteric nervous system through the release of serotonin and activation of serotonin-receptors (5-HT_4_) suggesting the strong interaction between the gut microbiota and the enteric nervous system and the potential role of both gut microbiota and serotonin in various gut diseases [89]. Gut microbiota influences the production of central BDNF levels that, in turn, regulates the maintenance of NMDAR production (receptors for N-methyl-D-aspartate), which are involved in synaptic plasticity and cognitive function. Dysbiosis of the gut microbiota results in a reduction of NMDAR input onto GABA inhibitory interneurons resulting in disinhibition of glutamatergic output because of which aberrant synaptic behaviour and cognitive deficits occur. These results imply that gut microbiota modulates BDNF function in the CNS, via changes in neurotransmitter function by altering the kynurenine pathway at least, in part, due to alterations in the availability and actions of SCFAs in the brain [90–97]. Since BDNF has anti-obesity and anti-diabetic actions, this explains the role of gut microbiota in the pathobiology of obesity, diabetes mellitus, and metabolic syndrome [97–103].

As already discussed above [69–73], EFAs influence both the gut microbiota proliferation and metabolism and serotonin formation and function [41–43, 104–106]. Thus, the gut microbiota, serotonin and EFAs function as one closely interlinked feedback regulatory system. These results imply that alterations in the dietary intake of EFAs alter gut microbiota and that, in turn, modulates maturation of enteric nervous system through serotonin synthesis and action. Gut microbiota can use EFAs to form SCFAs that mediate many of their beneficial actions.

### Conclusions and therapeutic implications

**Fig. 4:**
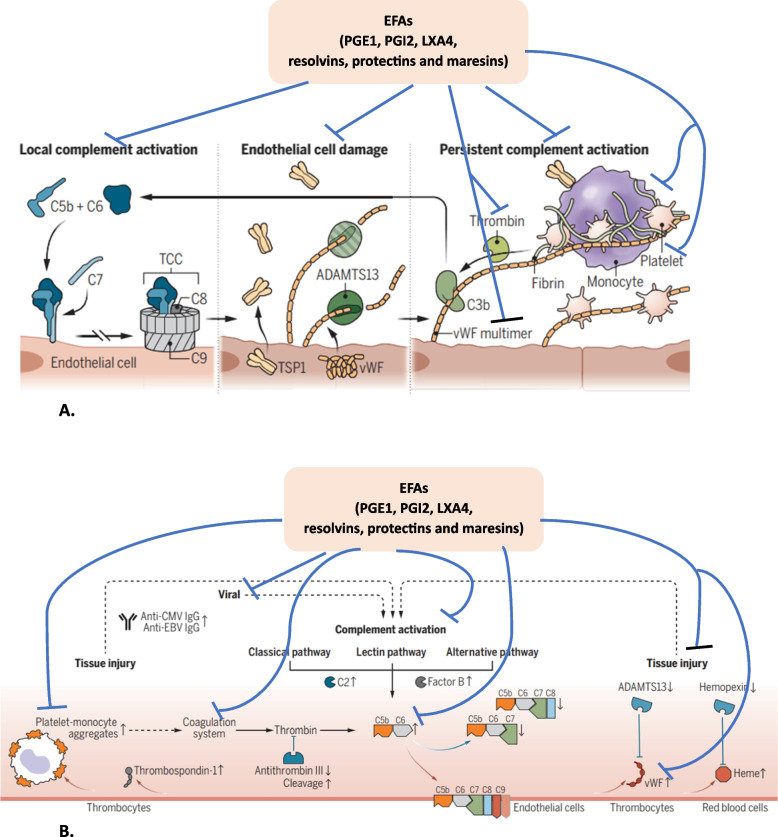
**A** Mechanisms by which EFAs and their beneficial metabolites prevent complement = coagulation and endothelial cell damage and useful in the prevention of long-haul syndrome due to COVID-19. Figure 4 **B** EFAs and their metabolites prevent complement activation, platelet-monocyte aggregation formation and tissue injury by virtue of their ability to maintain endothelial integrity, preventing platelet and monocyte activation, cytoprotective actions and virus inactivation. Figure 4**A** and **B** are modified from references [114, 115]

It is evident from the preceding discussion and evidence presented elsewhere [51–54, 107–110] that dietary EFAs have a significant role in the pathobiology of COVID-19 both directly and indirectly and the development of long-COVID or long-haul syndrome. It is proposed that insufficient intake of EFAs (LA and ALA) or formation of their long-chain metabolites (especially GLA, DGLA, AA, EPA, and DHA) due to a deficiency/genetic polymorphism of desaturases and elongases may render an individual susceptible to SARS-CoV-2 and other related viruses. This results in alteration of gut microbiota to induce deficiency in the maturation of gut enterochromaffin cells that leads to the excessive production of pro-inflammatory eicosanoids/cytokines resulting in decreased formation of serotonin and affecting its activity and subsequently develop the long haul or long COVID disorders. Less consumption of EFAs tends to exacerbate the pro-inflammatory cytokine production, as a consequence it activates the negative feedback control pathways for its metabolites. It is known that various viruses (especially SARS-CoV-2) interfere with the activity of desaturases that are essential for the conversion of dietary LA and ALA to their respective long-chain metabolites GLA, DGLA, AA and EPA and DHA (see Figs. 2 and 3) and the subsequent formation of their respective bioactive eicosanoids. SARS-CoV-2 activates phospholipase A2 (PLA2) that results in the release of GLA, DGLA, AA, EPA and DHA from the cell membrane lipid pool, and COX (cyclo-oxygenase) and LOX (lipoxygenase) enzymes that convert DGLA, AA, EPA and DHA to their respective PGs, LTs, TXS (thromboxanes) that have pro-inflammatory actions and LXA4 (from AA), resolvins (from EPA and DHA) and protectins and maresins (from DHA) that have vasodilator, platelet anti-aggregator, and anti-inflammatory actions. Activation of PLA2 and suppression of desaturases and elongases by SARS-CoV-2 results in the formation of excess of pro-inflammatory products from DGLA, AA, and EPA that promote profound pro-inflammatory events and hypotension, and ARDS (adult respiratory syndrome) in severe COVID-19. LXA4, resolvins, protectins and maresins are not only anti-inflammatory compounds but also suppress inappropriate immune activation. Thus, the balance between pro- and anti-inflammatory eicosanoid formed from DGLA, AA, EPA and DHA determine the severity of inflammation and immunosuppression. In the initial stages of SARS-CoV-2 infection, release of adequate amounts of LA, GLA, DGLA, AA, EPA and DHA are essential to inactivate the virus and produce the much-needed inflammation. But, once the inflammatory process has reached its peak, it is critical that adequate amounts of LXA4, resolvins, protectins and maresins are generated to suppress inappropriate inflammation and immune response so that anti-inflammatory events (especially resolution of inflammation), removal of debris from the site(s) of infection and wound healing occurs in a timely fashion to retore homeostasis. [111–113]. In the absence of such timely inflammation *vs* anti-inflammation and immune activation *vs* immunosuppression events as a result of excess activation of PLA2 and deficiency of desaturases and elongases enzymes would lead to an imbalance in the pro- vs anti-inflammatory eicosanoids and cytokines that is expected to result not only in the development of severe COVID-19 events, but can also flare the occurrence of long-COVID/long-haul syndrome later on because of continued inflammatory process and failure to eliminate virus/virus particles. This concept is supported by the recent report that during acute COVID-19 the complement and coagulation systems are activated and may remain activated in various tissues in long COVID (long haul) patients. Endothelial damage occurs because of insertion of complement C5b-C7 complex that induces the release of vWF (von Willebrand factor) and TSP1 (thrombospondin 1) that results in thrombotic events due to recruitment of platelets and monocytes. These and other related events such as reduced ADAMTS13 (the ADAMTS13 enzyme cuts vWF) to regulate the interaction with platelets. By regulating and proteolysis of vWF glycoprotein, the enzyme ADAMTS13 prevents triggering the formation of blood clots due to the formation and accumulation of ultra large vWF multimers that induces their binding to C3b resulting in complement activation via alternate pathway [114, 115]. These series of events occur because of damage to the vascular endothelial cells. EFAs and their metabolites (such as PGE1, PGI2, LXA4, resolvins, protectins and maresins) are needed for the integrity and normal function of vascular endothelial cells and prevent inappropriate activation of coagulation system. This argument is supported by the report that a reduction in the levels of sTM (thrombomodulin) and vWF occurs with n-3 fatty acids supplementation indicating an improvement in the hemostatic markers of endothelial dysfunction [116–120], and see Fig. 4), Thus, the concentrations of EFAs in the endothelial cell membrane is critical to endothelial cell health and integrity and prevention of thromboembolic events [120]. Based on the evidence(s) presented here, it is suggested that subclinical cell/tissue deficiency or inadequate intake of dietary LA and ALA (EFAs) and their inefficient conversion to AA, EPA and DHA and formation of their respective pro- and anti-inflammatory metabolites in a timely fashion can result in increased susceptibility to SARS-CoV-2, decreased formation of serotonin, defective immune response and ineffective wound healing resulting in the occurrence of post-COVID long haul syndrome. Hence, it is proposed that administration of adequate amounts of LA, GLA, DGLA, AA, EPA and DHA could be of benefit in the management of SARS-CoV-2 infection, and in the prevention and management of long haul or long CVOID.

## Data Availability

No datasets were generated or analysed during the current study.
